# The use of late preterm antenatal corticosteroids in women with gestational diabetes : a puzzle worth solving

**DOI:** 10.1186/s12884-024-06510-2

**Published:** 2024-04-18

**Authors:** Sadullah Özkan, Murat Levent Dereli, Dilara Kurt, Ahmet Kurt, Sadun Sucu, Fahri Burçin Fıratlıgil, Fatih İşleyen, Şevki Çelen, Yaprak Engin Üstün

**Affiliations:** 1 Department of Obstetrics and Gynecology, Division of Perinatology, Ankara Etlik Lady Zübeyde Maternity and Women’s Health Teaching and Research Hospital, P.O. box 06010, New Etlik Street No:55 Etlik, Keçiören, Ankara Turkey; 2 Department of Neonatology, Etlik Lady Zübeyde Maternity and Women’s Health Teaching and Research Hospital, Ankara, Turkey

**Keywords:** Antenatal corticosteroids, Gestational diabetes mellitus, Hyperbilirubinemia, Late preterm, Neonatal outcome

## Abstract

**Background:**

To investigate the association between late preterm antenatal corticosteroid treatment and outcome in late preterm neonates born to mothers with gestational diabetes mellitus,

**Methods:**

All patients with gestational diabetes mellitus who had a late preterm delivery at Etlik Lady Zübeyde Hospital between 2017 and 2021 were included. Women who met the inclusion criteria and were not given antenatal corticosteroid treatment during current pregnancy before 34 0/7 weeks of gestation were divided into two groups according to whether or not they received late preterm antenatal corticosteroid treatment. The two groups were compared in terms of adverse neonatal complications. The main outcomes were composite respiratory outcome and composite neonatal outcome. Logistic regression analysis was used to determine additional potential predictors of neonatal outcome.

**Results:**

This retrospective cohort study included a total of 400 participants with gestational diabetes mellitus who had a late preterm delivery within the study period. Of these women, 196 (49%) received late preterm antenatal corticosteroid treatment. Main outcomes showed no difference. Decreasing gestational age at birth was identified as an independent risk factor predicting both composite respiratory outcome and composite neonatal outcome in multivariate logistic regression analysis.

**Conclusions:**

Antenatal corticosteroid treatment at or after 34 0/7 weeks of gestation in women with gestational diabetes mellitus who had a late preterm delivery was not associated with improvement in adverse neonatal outcomes. Decreasing gestational age at birth was the only independent risk factor predicting composite neonatal and composite respiratory outcomes.

## Background

Any live birth before 37 completed weeks of gestation (WG) is defined as preterm birth (PTB), which is a leading cause of neonatal death and morbidity due to complications associated with prematurity, especially pulmonary hypoplasia [1]. Approximately one million infants die each year from complications of prematurity [2]. There are subcategories of PTB based on gestational age, including mild or late preterm (34–36 6/7 weeks), moderate preterm (32–33 6/7 weeks), very preterm (28–31 6/7 weeks), and extreme preterm (less than 28 weeks) [3]. Late preterm births account for 3–6% of all live singleton births and 65–75% of all live preterm births [4, 5]. Most clinical studies have focused primarily on preterm infants born before 34 weeks’ gestation who are more likely to have severe complications of prematurity due to PTB.

Antenatal corticosteroid treatment (ACT) to promote fetal lung maturation has long been recommended for pregnant women at risk of preterm delivery between 24 0/7 and 33 6/7 WG [6–9]. In these preterm neonates, complications including respiratory distress syndrome (RDS), intracranial hemorrhage, and necrotizing enterocolitis (NEC) occurred less frequently and with less severity, and the mortality rate was lower than in those whose mothers did not receive ACT [10, 11]. Despite lower morbidity and mortality rates in late preterm infants compared with those born at lower weeks, neonatal complications and mortality rates are much higher than in term infants [12, 13]. Besides, due to the adverse effects of fetal hyperglycemia and hyperinsulinemia on surfactant production, ACT may be even more important for reducing respiratory complications in neonates of diabetic mothers, regardless of the duration of pregnancy or type of diabetes [14, 15]. In many studies, ACT in the late preterm period resulted in a statistically significant reduction in neonatal respiratory complications, whereas in some older studies this reduction was not significant [16–21]. However, there are no conclusive data for the use of ACT in women with gestational diabetes mellitus (GDM) who delivered at 34 0/7 to 36 6/7 weeks’ gestation. There is a need for studies to bridge the gap between knowledge and practice in antenatal use of corticosteroids to reduce morbidity and mortality in these newborns. Besides, there are so many questions as to the side effects and benefits of ACT are yet to be clarified. Therefore, in this study, we aimed to compare neonatal outcomes between late preterm neonates born to mothers with GDM who were exposed to ACT and those who were not.

## Methods

### Study design

We conducted a retrospective cohort study of all women with GDM who experienced late preterm delivery between January 1, 2017, and December 31, 2021, in the high-risk pregnancy unit of Etlik Lady Zübeyde Maternity and Women’s Health Education and Research Hospital in Ankara, Turkey. The Ethics Committee of University of Health Sciences Etlik Lady Zübeyde Maternity and Women’s Health Education and Research Hospital, Ankara, Turkey, approved the conduct, protocol and procedures of the study (21.04.2022/90057706-799) and waived the need for informed consent to participate. After approval, the patients’ medical records were retrospectively reviewed.

### Characteristics of study population, patient selection and definitions

The inclusion criteria were gestational diabetics with singleton pregnancies who gave birth between 34 0/7 and 36 6/7 weeks of gestation. Patients with congenital fetal infections, fetal malformations and genetic anomalies (5), preterm premature rupture of membranes (29), premature rupture of membranes (24), patients who had received ACT before 34 0/7 WG during the current pregnancy (98), and patients with missing data (51) were excluded. In addition, patients in whom GDM was treated by diet alone (A1GDM) were included in the study, while patients who required medications (76) were excluded. All pregnant women who participated in the study had a reassuring fetal status (a maximum vertical pocket measurement of the amnion of ≥ 2 cm and a reactive non-stress test) on admission to the hospital. Cases with a reassuring antepartum fetal status at the time of hospital admission but requiring emergency delivery due to a non-reassuring intrapartum fetal status were included in the study, while patients already diagnosed with antepartum fetal distress at the time of admission (17) were excluded. After applying the exclusion criteria, 400 subjects were enrolled in the study, of whom 196 received ACT after 33 6/7 WG (treatment group) and 204 who did not receive ACT (control group). Maternal characteristics, pregnancy and neonatal outcomes were then compared.

The diagnosis of GDM was made using the criteria outlined in the American College of Obstetricians and Gynecologists (ACOG) guidelines using a two-step testing procedure between 24 and 28 WG. GDM was diagnosed if two or more serum glucose levels were outside the normal range defined in the Carpenter and Coustan diagnostic thresholds for the three-hour 100-g oral glucose tolerance test (fasting glucose level: 95 mg/dl; one-hour glucose level: 180 mg/dl; two-hour glucose level: 155 mg/dl; three-hour glucose level: 140 mg/dl) after a positive one-hour 50-g oral glucose challenge test (one-hour glucose level ≥ 140 mg/dl) [22].

Gestational age was calculated using the first day of the last menstrual period (LMP) and confirmed with sonographic dating. If the two calculations differed by less than or equal to seven days, the dating was based on the first day of the LMP. If the difference was more than seven days, sonographic dating was used. The delivery of a newborn between 34 0/7 and 36 6/7 WG is referred to as “late preterm birth” by the American Academy of Pediatrics (AAP), the ACOG, and the National Center for Health Statistics (NCHS) [23–25].

In our study, ACT was defined as steroid treatment in which delivery occurred at least 48 h after the first dose of two 12-mg doses of betamethasone administered intramuscularly 24 h apart with the indication for late preterm labor. The presence of uterine contractions of sufficient frequency and intensity to induce progressive cervical effacement and/or dilatation, detected by fetal cardiotocography and/or felt by manual palpation between 34 0/7 and 36 6/7 WG, is defined as late preterm labor.

Patients who underwent cesarean section immediately after the decision to deliver without waiting at least 6 h of fasting for safe anesthesia were defined as emergency cesarean sections, while others were defined as elective cesarean sections. The overall indications for cesarean section were as follows: fetal distress (7), previous cesarean Sect. (206), chord prolapse (1), preeclampsia with severe features (4), placental abruption (2), placenta previa (7), vaginismus (2), malpresentation (27), macrosomia (7), obstructed labor (12), dystocia (10), and failed induction of labor (7).

### Data collection

All data were obtained from the hospital database and medical records. Demographic characteristics such as maternal age, body mass index (BMI), comorbidities, gravidity, parity, gestational age at delivery, pregnancy complications, mode of delivery, previous abortions, previous cesarean deliveries, and preterm deliveries; fetal biometric measurements and maximum vertical pocket of amniotic fluid on sonography; Neonatal outcomes including appearance, pulse, grimace activity and respiration (APGAR) scores, birth weight, hypoglycemia, hyperbilirubinemia, sepsis, intraventricular hemorrhage (IVH), NEC, transient tachypnea of the newborn (TTN), RDS, need for mechanical ventilation, continuous positive airway pressure (CPAP) therapy, and neonatal intensive care unit (NICU) admission were assessed and compared between the two groups.

Because no neonatal death was observed in either group and due to the low incidence of serious neonatal adverse events in late preterm births, we use composite outcome measures that combine different components of neonatal outcome to draw conclusions. In addition, there were no serious neonatal adverse events, including sepsis, APGAR scores of less than 7 at 5 min, meconium aspiration syndrome, IVH, and NEC; main neonatal outcomes and measures were defined as composite neonatal outcome (CNO) consisting of any of the following that may result in serious neonatal morbidity: NICU admission, composite respiratory outcome (CRO) (any of the following: TTN, RDS, need for mechanical ventilation, and CPAP therapy), hypoglycemia, and hyperbilirubinemia.

### Statistical analysis

All statistical analyzes were performed using the RStudio integrated development environment for statistical computing (Affero General Public License v3; published 2011. RStudio for Linux, version v2021.09.4 + 403.pro3 Ghost Orchid; September 19, 2022; developed by Posit, PBC.) to analyze the data. Variables were examined using visual (histogram, probability plots) and analytic methods (Kolmogrov-Simirnov/Shapiro-Wilk test) to determine whether or not they were normally distributed. Descriptive analyzes were presented using means and standard deviations for normally distributed variables and using medians and quartiles (Quartile 1 - Quartile 3) for numerical data that were not normally distributed. Levene’s test was used to assess homogeneity of variance. To compare these parameters between groups, the Man-Whitney U test was performed. Descriptive analyzes were performed for the categorical variables using frequency and percentage. Relationships between categorical variables were analyzed using the chi-square test or Fisher’s exact test (when the assumptions of the chi-square test do not apply because of low expected cell counts). For the multivariate analysis, the possible factors identified in the univariate analyzes were entered into binary logistic regression analysis to identify additional independent predictors for CRO and CNO. The Hosmer-Lemeshow test was used to assess the fit of the logistic regression model. A type I error level of 5% was used to derive statistical significance. A p value of less than 0.05 was accepted as statistically significant.

## Results

**Fig. 1:**
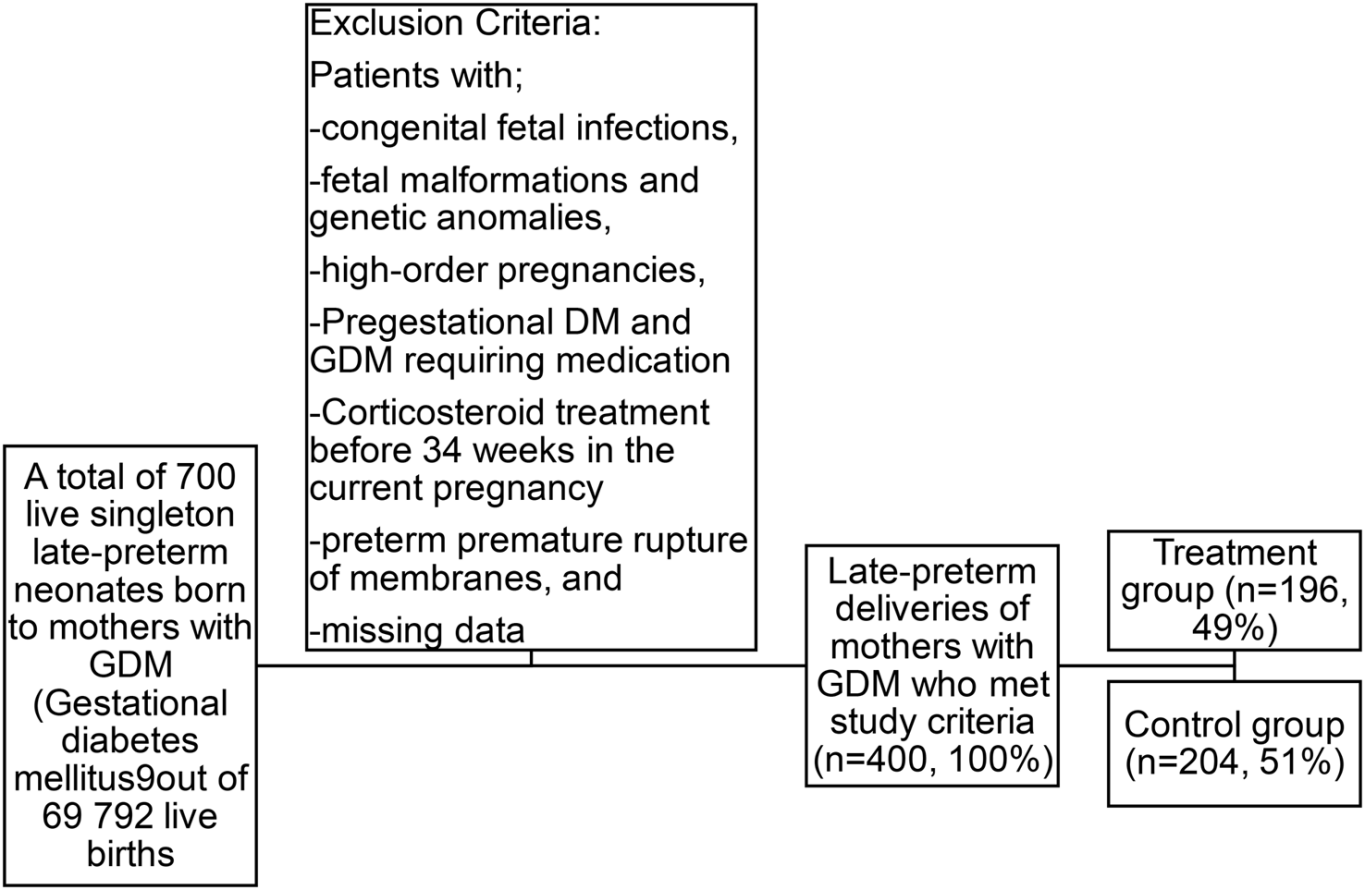
Flowchart of the study groups

This retrospective cohort study included a total of 400 participants with GDM who delivered between 34 0/7 and 36 6/7 WG during the study period. Of these, 196 (49%) women received late preterm ACT and 204 (51%) did not (Fig. 1). The demographic and clinical characteristics of the two groups are shown in Table 1.

**Table 1:** Demographics and basic clinical characteristics of patients in study groups

Variable	Treatment group (*n*: 196)	Control group (*n*:204)	*p*
Maternal age (years)	33 (28–37)	33 (29–37)	0.38
BMI (kg/m^2^)	31 (26–36)	32 (28–36)	0.09
Gravidity (number)	3 (2–4)	3 (2–5)	0.01^*^
Parity (number)	1 (1–2)	2 (1–2)	0.02^*^
Abortus (number)	0 (0–1)	0 (0–1)	0.65
Previous C/S (number)	0 (0–1)	0 (0–1)	0.14
Previous preterm delivery	42 (21.4)	42 (20.6)	0.83
Hypertensive disorders	41 (20.9)	36 (17.6)	0.40
Fetal growth restriction	17 (8.7)	19 (9.3)	0.96
Oligohydramnios	12 (6.1)	17 (8.3)	0.51

BMI, body mass index; C/S, cesarean section; kg/m^2^, kilograms per square meter

Data are expressed as median (Q1-Q3), or number (percentage) where appropriate. A p value of < 0.05 indicates a significant difference. Statistically significant p-values are indicated by the superscript asterisks. N is the total sample size consisted of patients

Characteristics including maternal age, BMI, number of previous abortions and cesarean sections, history of a preterm delivery, hypertensive disorders, fetal growth restriction, and oligohydramnios were indifferent between the two groups. The median numbers of gravidity and parity were significantly lower in the treatment group than in the control group [3 (2–4) vs. 3 (2–5), *p* = 0.01 and 1 (1–2) vs. 2 (1–2), *p* = 0.02, respectively)].

Analysis of neonatal characteristics and early neonatal outcomes of the study groups is shown in Table 2. Gestational age at birth, cesarean delivery, and female gender were indifferent between the two groups. In addition, there were no significant differences in terms of birthweight, small (SGA) and large for gestational age (LGA) newborns. The rate of hyperbilirubinemia was significantly higher in the treatment group (18.4% vs. 10.3%, *p* = 0.02). No significant differences were found in relation to CNO and CRO as the main outcomes defined in the Material and Methods section.

**Table 2:** Neonatal characteristics and early neonatal outcomes of the study groups

Variable	Treatment group (*n*:196)	Control group (*n*:204)	*p*
**Gestational age at birth (weeks)**			
34–35	46 (23.5)	52 (25.5)	0.61
35–36	71 (36.2)	52 (25.5)	
36–37	79 (40.3)	100 (49)	
**Cesarean delivery**	140 (71.8)	152 (74.5)	0.54
Urgent	39 (27.9)	29 (19.1)	0.08
Elective	101 (72.1)	123 (80.9)	
Female gender	79 (40.3)	83 (40.7)	0.93
**Birthweight (grams)**			
< 2500	41 (20.9)	28 (13.7)	0.05
≥ 2500	155 (79.1)	176 (86.3)	
Small for gestational age	23 (11.7)	19 (9.3)	0.43
Large for gestational age	36 (18.4)	54 (26.5)	0.05
APGAR 1	9 (8.25-9)	9 (8–9)	0.55
APGAR 5	10 (10–10)	10 (10–10)	0.38
NICU admission	61 (31.1)	47 (23)	0.06
Hypoglycemia	8 (4.1)	15 (7.4)	0.16
Hyperbilirubinemia	36 (18.4)	21 (10.3)	0.02^*^
CPAP	14 (7.1)	6 (2.9)	0.09
TTN	1 (0.5)	2 (1)	> 0.05
RDS	8 (4.1)	12 (5.9)	0.55
Mechanical ventilation	15 (7.7)	19 (9.3)	0.67
Composite respiratory outcome	25 (12.8)	24 (11.8)	0.88
Composite neonatal outcome	61 (31.1)	48 (23.5)	0.08

APGAR, appearance, pulse, grimace, activity, and respiration score; CPAP, continuous positive airway pressure; NICU, neonatal intensive care unit; RDS, respiratory distress syndrome; TTN, transient tachypnea of the newborn

Data are expressed as median (Q1-Q3), or number (percentage) where appropriate. A p value of < 0.05 indicates a significant difference. Statistically significant p-values are indicated by the superscript asterisks. n is the total sample size consisted of newborns

Gestational age at birth was identified as an independent factor predicting both CRO [Odds ratio (OR) = 0.36, 95% Confidence Interval (CI) 0.25–0.51, *p* < 0.01) and CNO (OR = 0.403, 95% CI 0.310–0.526, *p* < 0.01)] in multivariate logistic regression analysis. Birthweight, female gender and mode of delivery were not found to be independent risk factors for either CRO or CNO. Late preterm ACT found to have no effect on both CRO and CNO (Table 3). Each 1 week increase in gestational age at birth resulted in 2.7- and 2.48-fold protection from the adverse results of CRO and CNO, respectively.

**Table 3:** Multivariate logistic regression analysis of risk factors for composite respiratory and neonatal outcomes

Variable	Composite respiratory outcome		Composite neonatal outcome	
	**OR (95% CI)**	***p***	**OR (95% CI)**	***p***
GAAB	0.36 (0.25–0.51)	< 0.01^*^	0.403 (0.310–0.526)	< 0.01^*^
Birthweight	1 (0.999–1.001)	0.54	1 (0.999-1)	0.28
Female gender	0.559 (0.275–1.139)	0.10	0.774 (0.474–1.264)	0.30
Vaginal delivery^#^	0.426 (0.171–1058)	0.07	0.719 (0.396–1.306)	0.28
No ACT	0.912 (0.502–1.658)	0.76	0.681 (0.437–1.060)	0.09
No ACT^¥^	1.123 (0.598–2.108)	0.72	0.860 (0.539–1.372)	0.53
No ACT^§^	1.214 (0.638–2.311)	0.55	0.866 (0.542–1.384)	0.55

ACT, antenatal corticosteroid treatment; CI, confidence interval; GAAB, gestational age at birth; OR, relative risk

Data are expressed as median (minimum-maximum). A p value of < 0.05 indicates a significant difference. Statistically significant p-values are indicated by the superscript asterisks

^#^: Antepartum and intrapartum fetal distress indicated cesarean delivery excluded

^¥^: Female gender and birthweight adjusted

^§^: Female gender, birthweight, maternal age and body mass index adjusted

## Discussion

The main findings of our study were: (1) Antenatal corticosteroids administered between 34 0/7 and 36 6/7 WG were not significantly associated with a reduction in adverse CNO, CRO, or any of the individual neonatal complications in women with GDM who delivered late preterm; (2) Neonatal birthweight was indifferent, and SGA and LGA rates also showed no significant differences between the two groups; (3) Late preterm ACT was significantly associated with increased rates of hyperbilirubinemia in neonates born late-preterm to mothers with GDM; (4) Gestational age at birth was identified as an independent risk factor predicting both CRO and CNO.

In recent decades, obesity has become a growing problem worldwide. As a result of rising obesity rates due to unhealthy and wrong eating habits and the fact that women become pregnant at a later age, diabetes has increased during pregnancy. In addition, there is a study showing that GDM has a genetic background and that the risk of developing GDM can be determined using a genetic risk score [26].

Maternal administration of corticosteroids in women in whom preterm delivery is inevitable is one of the most important prenatal interventions to improve neonatal outcome, particularly RDS [27–29]. The use of a single course of corticosteroids is recommended for pregnant women between 24 0/7 and 33 6/7 WG and may be considered for pregnant women between 23 0/7 and 24 0/7 WG who are at increased risk for preterm delivery within 7 days, regardless of fetal membrane status, whereas no significant difference in perinatal outcomes was observed between 22 0/7 and 22 6/7 WG [11, 27, 30–32]. Interestingly, recent data suggest that ACT may be beneficial in women at risk of imminent late PTB who have not given a prior course of antenatal corticosteroids during their current pregnancies [29]. There was no increased risk of neonatal sepsis, chorioamnionitis, or endometritis with corticosteroids given during the late preterm period, whereas hypoglycemia was more common in infants exposed to betamethasone, although no adverse events were reported in this context [33].

In a retrospective study by Krispin et al., similar results were found on neonatal outcomes. However, in contrast to our study, the patients in the treatment group consisted of women who were given antenatal corticosteroids between 24 0/7 and 33 6/7 WG, and some of them delivered their babies in the late preterm period, whereas others were delivered in the term period [34]. Fetal macrosomia resulting from fetal hyperinsulinemia in response to maternal diabetes and antenatal corticosteroids is the expected outcome. However, in our study, which is consistent with the results of the abovementioned study, no differences in SGA and LGA rates were observed between the two groups. We think that this result might be related to the similar gestational age at birth in the treatment group compared to the control group.

While ACT reduces the risk of neonatal morbidity and mortality, the resulting maternal hyperglycemia may also cause other neonatal effects, particularly neonatal hyperglycemia. Increased hepatic gluconeogenesis along with decreased glucagon levels, and decreased peripheral glucose uptake are the main mechanisms for maternal hyperglycemia induced by ACT [35]. In contrast to the long-lasting beneficial effects of ACT on the preterm infant, the maternal hyperglycemia induced by corticosteroids lasts only a few days [36]. Therefore, this effect is often negligible in pregnant women with diabetes, especially with good glycemic control, and is not a barrier to ACT. Moreover, the risk of resulting neonatal hypoglycemia is not common unless delivery occurs within a few days of steroid administration. In our study, neonatal hypoglycemia rates were low and no significant difference was observed between the two groups. We think that the low rates of hypoglycemia in neonates of mothers treated with corticosteroids may be related to the fact that the births occurred at least 48 h after the first dose, a time interval when the intensity of steroid-induced maternal hyperglycemia is greatest. Because neonates born before 48 h after the first corticosteroid dose were not included in our study and protective mechanisms against hypoglycemia function better in late preterm infants than in those born at earlier WG, we observed similar neonatal hypoglycemia rates between the two groups.

Furthermore, ACT may cause neonatal hyperbilirubinemia as another important metabolic complication [35, 37]. The presumed causal theory for hyperbilirubinemia is that dexamethasone and betamethasone may decrease hepatic uptake of unconjugated bilirubin by acting as competitive inhibitors [38]. While there was no difference between the groups in terms of neonatal hypoglycemia, we found that neonatal hyperbilirubinemia was significantly higher in the treatment group, which is consistent with the literature.

Our study has some limitations, mainly due to its retrospective nature. The major strength of this study is the ability to analyze a specific population that has not yet been thoroughly studied on this topic. In addition, to the best of our knowledge, this article represents the first study to examine the use of late preterm antenatal corticosteroids in neonates born late-preterm to mothers with GDM. Furthermore, the study was conducted in a single tertiary medical center with a high patient volume, where the standardized algorithms for the diagnosis, treatment, and follow-up of GDM were applied.

## Conclusions

In conclusion, our study showed no improvement in outcomes in neonates born late-preterm to mothers with GDM when ACT was administered in the late preterm period. In addition, decreasing gestational age at birth was found to be the only independent risk factor predicting neonatal outcome. Further prospectively designed randomized controlled trials are needed to better define the efficacy, safety, and limitations of ACT in diabetic women at high risk for late preterm delivery.

## Data Availability

The datasets used and/or analysed during the current study are available from the corresponding author on reasonable request.

## Abbreviations

AAP: American Academy of Pediatrics
ACOG: American College of Obstetricians and Gynecologists
ACT: Antenatal corticosteroid treatment
APGAR: Appearance, pulse, grimace activity and respiration
BMI: Body mass index
CI: Confidence Interval
CNO: Composite neonatal outcome
CPAP: Continuous positive airway pressure
CRO: Composite respiratory outcome
GDM: Gestational diabetes mellitus
IVH: Intraventricular hemorrhage
LGA: Large for gestational age
LMP: Last menstrual period
NCHS: National Center for Health Statistics
NEC: Necrotizing enterocolitis
NICU: Neonatal intensive care unit
OR: Odds ratio
PTB: Preterm birth
RDS: Respiratory distress syndrome
SGA: Small for gestational age
TTN: Transient tachypnea of the newborn
WG: Weeks of gestation
